# Lysophosphatidic acid plasma concentrations in healthy subjects: circadian rhythm and associations with demographic, anthropometric and biochemical parameters

**DOI:** 10.1186/s12944-017-0536-0

**Published:** 2017-07-21

**Authors:** Anna Michalczyk, Marta Budkowska, Barbara Dołęgowska, Dariusz Chlubek, Krzysztof Safranow

**Affiliations:** 1 Department of Biochemistry and Medical Chemistry, Pomeranian Medical University in Szczecin, 70-111 Szczecin, Poland; 2 Department of Medical Analytics, Pomeranian Medical University in Szczecin, 70-111 Szczecin, Poland; 3 Department of Microbiology, Immunology and Laboratory Medicine, Pomeranian Medical University in Szczecin, 70-111 Szczecin, Poland

**Keywords:** Lysophosphatidic acid, Lysophospholipids, Reference values, Antropometric parameters, Demographic parameters, Circadian rhythm, Healthy subjects

## Abstract

**Background:**

Lysophosphatidic acid (LPA) is a bioactive lipid with a wide biological activity. Previous studies have shown its potential usefulness as a diagnostic marker for ovarian cancer. The aim of the study was to investigate which factors may influence plasma LPA concentrations in healthy subjects and to propose reference values.

**Methods:**

The study group consisted of 100 healthy subjects. From all of them the blood samples were taken at 7 a.m. (fasting state). From 40 volunteers additional blood samples were taken at 2 p.m., at 8 p.m. and at 2 a.m. next morning. Concentrations of LPA were measured in plasma samples using enzyme-linked immunosorbent assay.

**Results:**

Analysis of samples from 100 healthy volunteers showed significant influence of sex and age on plasma LPA. The reference range for the plasma LPA concentration corrected for age and sex, determined at 2.5–97.5 percentile interval is 0.14–1.64 μM. LPA correlates positively with BMI, serum total cholesterol, triacylglycerols, uric acid and negatively with estimated glomerular filtration rate and serum albumin. Concentration of LPA at 2 a.m. was lower than at 2 p.m. There were not any significant differences between plasma LPA at 7 a.m. and any other time of the day.

**Conclusions:**

Plasma LPA is associated with demographic, anthropometric and biochemical parameters. It seems that LPA concentrations have no specific circadian rhythm and the time of donation and fasting state have marginal effect on plasma LPA. These findings may be helpful in future incorporation of LPA as a diagnostic marker.

## Background

Lysophosphatidic acid (LPA) is a bioactive lysophospholipid present both intra- and extracellularly. Intracellular LPA is an intermediate metabolite in the synthesis of other phospholipids. Extracellular LPA acts through specific G protein coupled receptors (GPCR) belonging to the endothelial differentiation gene (LPA1–3) or purinergic (LPA4–6) receptors family, which are present on the surface of numerous cell types [1]. Activation of LPA receptors results in enhanced proliferation, migration and invasiveness, inhibition of apoptosis, morphological changes, inhibition of differentiation, contraction, increased endothelial permeability, platelet aggregation and cytokine secretion. The effect of LPA administration is dependent on the activated receptor, type of the cell and molecular species of LPA [2, 3].

Despite its simple structure, LPA is important element of proper development and functioning of variety tissues and organ systems. It was shown, that LPA participates in development of nervous [4, 5] and circulatory [6] system, functioning of male and female reproductive system [7], bone metabolism [8], hair follicles development [9], wound healing [10] and functioning of immune system [7]. On the other hand abnormalities in LPA signaling are involved in cancer progression [11] atherosclerosis [3], neuropathic pain [12], neuropsychiatric disorders [1], autoimmune diseases [1] and renal and pulmonary fibrosis [13, 14].

The main sources of LPA in blood are other lysophospholipids, especially lysophosphatidylcholine (LPC) and the reaction of hydrolysis of lysophospholipids to LPA is catalyzed by autotaxin (ATX) and its activity of lysophospholipase D [15]. LPA is present in multiple body fluids, such as blood [16, 17], saliva [18] and urine [19]. Serum concentrations of LPA are higher than in plasma, which is probably caused by secretion of LPA by activated platelets [20].

There are several studies indicating that plasma LPA is promising diagnostic marker for ovarian cancer (for meta-analysis see [21]). LPA concentrations in ovarian cancer patients are elevated already in the early stages of the disease [17]. Other conditions related to increased LPA plasma concentrations are acute coronary [22], chronic hepatitis C [23] and renal failure [24]. Due to the potential usefulness of LPA as a diagnostic marker the aim of the study was to investigate which factors may influence plasma LPA concentrations in healthy subjects and to propose reference values. This knowledge may be helpful for proper interpretation of elevated LPA plasma concentrations.

## Methods

The study group consisted of 100 healthy volunteers from Szczecin in Poland. The intent of the study was to provide a baseline of LPA measurement as a diagnostic marker for ovarian (and possibly) other cancers, therefore in the study group “healthy” means cancer free, not necessarily free of any physical issues. The inclusion criterion was the age over 18 years old. The exclusion criteria were lack of the informed consent, pregnancy, diabetes and other metabolic diseases, acute and chronic inflammatory diseases and malignant neoplasms. The study had approval from the Local Bioethics Committee at the Pomeranian Medical University in Szczecin and written informed consent was obtained from all volunteers. Characteristics of the study group are presented in Tables 1 and 2.

**Table 1:** Baseline characteristics of the study group

Parameter		Total	Women	Men	p*
N		100	53	47	
Smoking (yes/no)		21/79	13/40	8/39	
Age [years]	Mean ± SD	39.6 ± 14.5	37.5 ± 14.8	41.9 ± 13.9	0.057
	Median	34.0	30.0	37.0	
Body mass [kg]	Mean ± SD	73.4 ± 14.9	65.2 ± 12.0	82.6 ± 12.3	<0.001
	Median	72.0	61.0	80.0	
BMI [kg/m^2^]	Mean ± SD	24.8 ± 4.0	24.0 ± 4.3	25.6 ± 3.4	0.009
	Median	24.0	22.7	25.5	

*Mann-Whitney test for difference between women and men

**Table 2:** A history of past/chronic diseases in the study group

Disease	Number
Arterial hypertension	6
Atherosclerosis	3
Varicose veins	7
A history of hypothyroidism	1
A history of bronchitis / pneumonia	10
A history of cholelithiasis	4
A history of gastric / duodenal ulcer	3
A history of urolithiasis	1

The blood samples were taken from all of the volunteers in the morning at about 7 a.m. (fasting state). Additional blood samples were collected at 2 p.m., 8 p.m., and 2 a.m. next morning from a subgroup of 40 volunteers (18 women and 22 men, mean age 33 ± 9 years, median 30 years). The blood samples were taken from a peripheral vein and placed in tubes containing ethylenediaminetetraacetic acid (K_2_EDTA) as anticoagulant and in tubes without anticoagulant and centrifuged (10 min, 1000 g, 20 °C) within half an hour from donation. In the case of an incomplete clotting in the sample without anticoagulant time to centrifugation was prolonged to one hour. Obtained plasma and serum samples were frozen and stored in −80 °C until used.

Basic biochemical parameters including total protein (TP), albumin, glucose, total cholesterol (TC), triacylglycerols (TAG), HDL-cholesterol (HDL-C) (precipitation method with phosphotungstic acid and magnesium chloride), and uric acid (UA) were measured in the serum samples using reagent kits from BioMaxima (Lublin, Poland). Absorbances of the samples were measured using a microplate reader EnVision 2104 Multilabel Reader (Perkin Elmer). LDL-cholesterol (LDL-C) concentrations were calculated from TC, HDL-C, and TAG using the Friedewald formula. The concentrations of serum creatinine were measured using a kit reagent from BioMaxima (Lublin, Poland). Kinetic measurements of absorbances were performed using the Specord 250 (Analytik Jena). Estimated glomerular filtration rate (eGFR) was calculated on the basis of age, gender and serum creatinine concentration using CKD-EPI formula [25]. Plasma lysophosphatidic acid concentrations were measured using competitive enzyme-linked immunosorbent assay (LPA Assay Kit II from Echelon Biosciences Inc., Salt Lake City, United States of America; Cat no. K-2800S). The absorbance was measured using microplate reader described above.

Statistical analysis was performed using Statistica 12 (StatSoft Inc.). The Shapiro-Wilk test was used to investigate the normality of distribution. Most of the variables were non-normally distributed, therefore the nonparametric tests were used to investigate the significance of differences between groups (Mann-Whitney U test for independent groups, Friedman ANOVA and Wilcoxon signed-rank test for comparisons of dependent measurements) and correlations between variables (Spearman’s rank correlation coefficient). General linear model (GLM) was used to analyze association of age and sex with LPA plasma concentration, which was transformed logarithmically to normalize its distribution. Differences were considered as statistically significant if *p*-values were less than 0.05.

## Results

LPA fasting plasma concentrations in healthy volunteers ranged from 0.06 to 3.46 μM. In the women subgroup they were significantly higher than in men (Table 3).

**Table 3:** LPA fasting concentrations in plasma samples of 100 healthy volunteers (μM)

	Number	Mean	SD	Minimum	Maximum	Lower quartile	Median	Upper quartile	p*
Total	100	0.89	0.60	0.06	3.46	0.49	0.74	1.12	
Women	53	1.00	0.66	0.06	3.46	0.60	0.83	1.17	0.026
Men	47	0.77	0.50	0.15	2.04	0.39	0.63	0.98	

*Mann-Whitney test for difference between women and men

The reference range at 2.5–97.5 percentile interval is 0.18–2.61 μM for females and 0.17–1.88 μM for males.

**Fig. 1:**
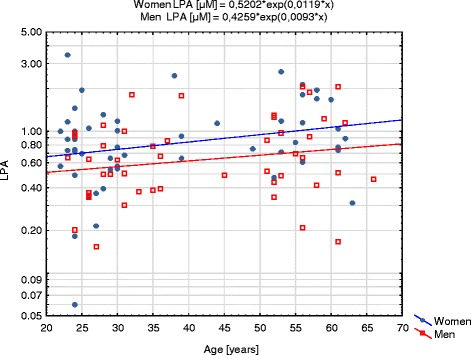
Correlation of LPA with age in women and men subgroups. LPA concentrations are presented with logarithmic scale

Univariate statistical analysis did not show significant correlation between LPA and age (Spearman’s *R* = 0.16, *p* = 0.12 in total group; *R* = 0.18 *p* = 0.20 in women and *R* = 0.22; *p* = 0.23 in men), but suggested that positive association may exist in both sex groups (Fig. 1). Multivariate analysis taking into account age and sex showed that both older age and female sex are significant independent predictors of higher LPA concentrations (Table 4). On the basis of GLM equation coefficients the following formula for calculation of LPA concentration adjusted for age and sex (LPA_adj_) was derived:

$$ {LPA}_{adj}\ \left[\mu M\right]={\mathrm{e}}^{\ln\ \left(LPA\ \left[\mu M\right]\right)-0.0108\ast \mathrm{age}\ \left[\mathrm{years}\right]+0.3068\ \left(\mathrm{if}\ \mathrm{male}\ \mathrm{sex}\right)} $$

**Table 4:** Multivariate analysis (GLM) of influence of age and sex on logarithm of LPA concentrations in the study group (*n* = 100)

Independent variables	Coefficient in GLM equation ± standard error	*P*
Intercept^a^	−0.6098 ± 0.1957	0.002
Male sex	−0.3068 ± 0.1338	0.024
Age [years]	0.0108 ± 0.0046	0.022

R^2^ = 0.087, *p* = 0.012 for the whole GLM

^a^Intercept value approximates the mean logarithm of LPA concentrations adjusted for females at age 0 and corresponds to 0.543 μM

The median (interquartile range) for the LPA_adj_ in our group of volunteers is 0.56 (0.38–0.80) μM and the reference range at 2.5–97.5 percentile interval is 0.14–1.64 μM.

**Fig. 2:**
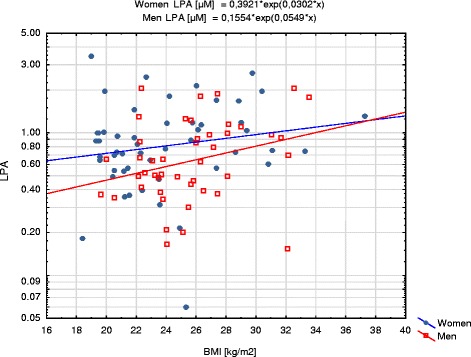
Correlation of LPA with BMI in women and men subgroups. LPA concentrations are presented with logarithmic scale

**Fig. 3:**
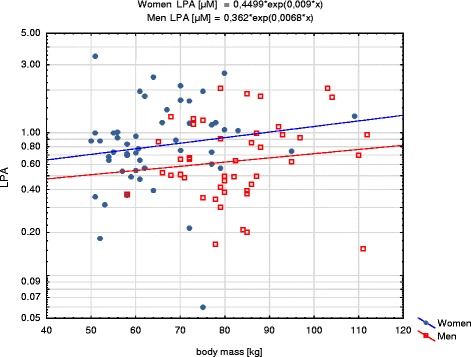
Correlation of LPA with body mass in women and men subgroups. LPA concentrations are presented with logarithmic scale

**Fig. 4:**
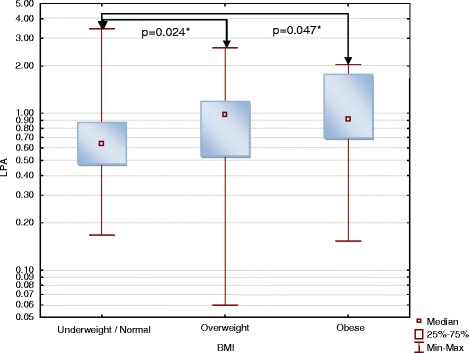
Association between LPA and BMI range. Underweight/normal – BMI <25; Overweight – BMI 25–29.99; Obese – BMI ≥30 kg/m^2^. LPA concentrations [μmol/L] are presented with logarithmic scale. * Wilcoxon signed-rank test

LPA showed statistically significant positive correlations with BMI in the whole group (*R* = 0.22; *p* = 0.034) and in men subgroup (*R* = 0.31; *p* = 0.041) (Fig. 2) and with body mass in women subgroup (*R* = 0.29; *p* = 0.041) (Fig. 3). Overweight and obese patients had significantly higher plasma LPA concentrations than patients with BMI <25 kg/m^2^ (*p* = 0.024 and *p* = 0.047, respectively, Fig. 4).

**Fig. 5:**
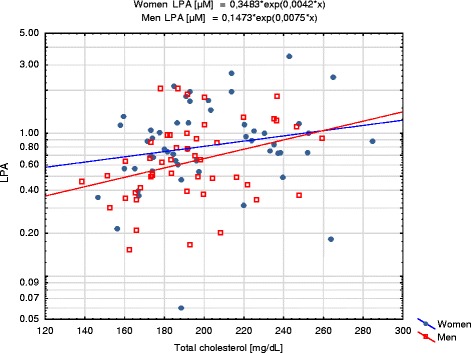
Correlation between LPA and total cholesterol. LPA concentrations are presented with logarithmic scale

**Fig. 6:**
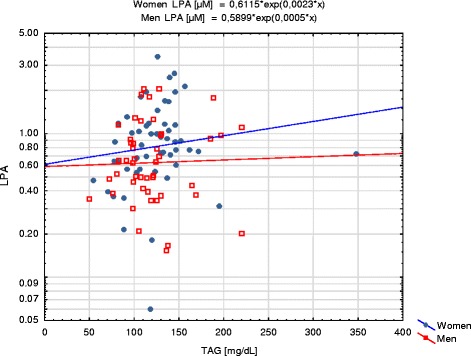
Correlation between LPA and triacylglycerols (TAG). LPA concentrations are presented with logarithmic scale

Among the lipid parameters LPA was positively correlated with TC (*R* = 0.32; *p* = 0.0011, Fig. 5) and TAG (*R* = 0.21; *p* = 0.0032, Fig. 6) in total group, TAG (*R* = 0.29; *p* = 0.036) in women and TC (*R* = 0.33; *p* = 0.026) in men subgroup. In the total group and men subgroup negative correlation between LPA and albumin was observed (*R* = −0.24; *p* = 0.016 and *R* = −0.30; *p* = 0.041, respectively). In men subgroup LPA was positively correlated with UA (*R* = 0.37; *p* = 0.011). In women subgroup LPA was negatively correlated with eGFR (*R* = −0.30; *p* = 0.029). No correlations were observed between LPA and total protein, glucose, creatinine and cholesterol of HDL or LDL fraction. Similar correlations with eGFR in women subgroup and with BMI, TC, UA and albumin in men subgroup were observed for LPA_adj_ (Table 5). However, only positive correlation with TC remained significant for LPA_adj_ in the whole study group. There were no statistically significant differences in LPA plasma concentrations between smokers (*N* = 21) and non-smokers (*N* = 71) (*p* = 0.91).

**Table 5:** Correlations between LPA and other parameters in the study group. Spearman rank correlations coefficients are presented for raw (LPA) and sex- and age-adjusted (LPA_adj_) concentrations

	Total			Women			Men		
	N	LPA	LPA_adj_	N	LPA	LPA_adj_	N	LPA	LPA_adj_
Age	100	0.156	−0.029	53	0.181	−0.071	47	0.224	−0.008
Body mass	96	0.032	0.136	51	0.287*	0.170	45	0.161	0.228
BMI	96	0.216*	0.185	51	0.240	0.078	45	0.306*	0.302*
Creatinine	100	−0.118	0.081	53	−0.001	0.190	47	−0.186	−0.047
eGFR	100	−0.170	−0.109	53	−0.302*	−0.308*	47	0.075	0.129
TC	100	0.320*	0.289*	53	0.261	0.219	47	0.326*	0.358*
LDL-C	100	0.190	0.177	53	0.210	0.214	47	0.092	0.140
HDL-C	100	0.105	0.034	53	−0.062	−0.180	47	0.284	0.214
TAG	100	0.214*	0.171	53	0.289*	0.202	47	0.022	0.071
Glucose	100	0.074	0.050	53	0.201	0.188	47	−0.101	−0.076
UA	100	0.019	0.058	53	−0.051	−0.197	47	0.367*	0.352*
TP	100	0.142	0.142	53	0.159	0.233	47	0.002	0.012
Albumin	100	−0.241*	−0.165	53	−0.082	−0.023	47	−0.299*	−0.291*

*p < 0.05

**Fig. 7:**
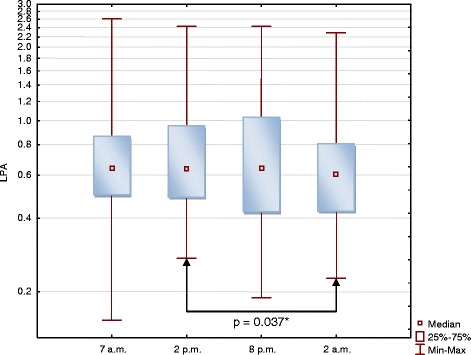
Circadian rhythm of plasma LPA concentrations in a subgroup of 40 subjects. LPA concentrations are presented with logarithmic scale. Friedman’s ANOVA *p* = 0.09. * Wilcoxon signed-rank test

In circadian rhythm analysis, LPA differed slightly depending on the time of the day, but this relationship did not reach statistical significance (Fig. 7). At 2 a.m. LPA concentrations were significantly lower than at 2 p.m. (*p* = 0.04). No significant differences between the samples taken at 7 a.m. and at any other time of the day were found.

## Discussion

LPA is promising candidate for diagnostic marker, but before its application in routine diagnostics many issues need to be taken into account. One of them is selection of proper material and method. In order to ensure conditions as close as possible to those used for other plasma parameters and most of the previous scientific studies on LPA, in the current study blood was drown from the peripheral vein to standard EDTA containing tubes and plasma samples were obtained by centrifugation of the samples at room temperature. In serum samples concentration of LPA grows much faster than in plasma during incubation, therefore serum samples are not recommended for LPA quantification [26]. According to Nakamura et al. [27] optimal material for LPA quantification is plasma obtained by centrifugation of whole blood samples collected to the tubes containing 7.5% EDTA with 10% CTAD (citrate, theophylline, adenosine, and dipyridamole) as an anticoagulant. This procedure inhibits in vitro LPA production to the greatest extent. However in cited study [27] using standard EDTA containing tubes did not cause significant increase in LPA concentrations after one hour incubation at room temperature and these blood collection tubes are commercially available.

The next step was the selection of optimal method. There are many methods for LPA quantification (for review see [28]). Some of them allow quantification of LPA molecular species and other only total LPA concentration in the samples. All of these methods differ in process of sample preparation, sensitivity, required equipment and time and labor consumption. The most widely studied method in scientific research is liquid chromatography with a double mass spectrometry (LC/MS/MS). The main disadvantage of this method is the necessity of possessing expensive equipment not available in most laboratories. Therefore we chose enzyme-linked immunosorbent assay (ELISA) as a method of LPA quantification, because of its relatively small equipment requirements and no need of prior lipid extraction which makes it possible to be used in routine diagnostics.

Mean LPA concentration obtained in the current study for healthy subjects was 0.89 ± 0.60 μM. This result was slightly higher than those obtained previously in healthy subjects with the use of methods based on liquid or gas chromatography, much higher than those obtained with the enzymatic assay and lower than in other assays (see Table 6). It should be noted, however, that these results are dependent not only on the measurement method and sample preparation procedures but also on the criteria of selection for the healthy subjects in the cited studies.

**Table 6:** Comparison of mean LPA concentrations obtained in healthy subjects in previous studies. W- women, M- men, GC – gas chromatography, LC – liquid chromatography, MS- mass spectrometry

Source	Method	Size of the group (N)	LPA (μM) Mean ± SD
Bese et al. [17]	GC/MS	50 (W)	0.6 ± 0.42
Scherer et al. [35]	LC/MS/MS	10	0.699 ± 0.2
Baker et al. [36]	LC/MS	10	M: 0.61 ± 0.14 W: 0.74 ± 0.17
Xu et al. [37]	GC	48 (W)	0.6 ± 0.19
Hosogaya et al. [29]	Enzymatic	146	M: 0.077 ± 0.026 W: 0.103 ± 0.032
Li et al. [38]	LC/ measurement of inorganic phosphorus	136	1.77 ± 1.04
Yao et al. [30]	Microcolumn/ measurement of inorganic phosphorus	670	2.57 ± 1.96
Sedláková et al. [39]	Capillary electrophoresis	27	3.1 (range 0.94–9.73)

In the current study significantly higher concentrations of LPA were observed in women than in men. These results are consistent with Hosogaya et al. study [29], but opposite to Yao study [30] in which no significant differences were observed. Higher LPA concentrations in women may results from higher lysophospholipase D activity in women than in men [31].

The influence of the age on LPA concentrations is controversial. Yao et al. [30] did not observe influence of the age on LPA concentrations. Hosogaya et al. [29] observed negative correlation between age and LPA in women and no correlation in total group. In the current study correlation between age and LPA did not achieve statistical significance in univariate analysis, but multivariate analysis showed that both older age and female sex are independent significant predictors of higher plasma LPA. Future studies are necessary to confirm and explain this relationship.

The study showed that overweight and obesity are associated with significantly higher plasma LPA. This relationship may results from the role of LPA in pathophysiology of obesity. ATX – LPA signaling axis is probably engaged in inhibition of adipose tissue expansion [32]. In human, obesity is associated with higher ATX expression in visceral adipose tissue [33]. Study of Dusaulcy et al. [34] on transgenic mice carrying a null ATX allele specifically in adipose tissue (FATX-KO) showed enhanced nutritional fattening and about 40% reduced plasma LPA compared to wild-type mice. This observation indicates the potential influence of adipose tissue on the plasma LPA concentration and may explain association of higher plasma LPA with overweight/obesity.

Obesity is often associated with dyslipidemia. Plasma LPA concentration was positively correlated with serum TC and TAG and the correlation with TC remained significant for sex- and age-adjusted LPA_adj_. This result is consistent with the Yao et al. study [30] showing that patients with hyperlipidemia (defined as TC ≥ 5.18 mmol/l or LDL-C ≥ 3.37 mmol/l) have higher LPA plasma concentrations than control group. Negative correlation of LPA with eGFR and positive correlation between LPA and UA may suggest its potential association with renal function. Mechanisms of above relationships are not known and remain to be clarified in future studies.

It seems that there is no specific circadian rhythm of LPA concentrations. The only observed significant difference was lower concentration at 2 a.m. at night than in the afternoon. There were no significant differences between samples in the morning at 7 a.m. in fasting state and samples taken at any of the other three time points. Therefore time of donation and prior fasting seem to have negligible influence on the measurement.

## Conclusions

In conclusion the reference range for the plasma LPA concentration corrected for age and sex (LPA_adj_), determined at 2.5–97.5 percentile interval is 0.14–1.64 μM. There are many factors associated with LPA plasma concentrations in healthy subjects. Women have higher plasma LPA concentrations than men; therefore sex should be taken into account in the establishing of reference LPA values. Age seems to be positively associated with LPA concentrations. Another important notice is association between LPA and BMI and/or body mass. Obese people may have higher plasma LPA concentrations and this factor should be considered during interpretation of assay results. Smoking probably does not influence LPA concentrations. Plasma LPA is positively correlated with serum TC, TAG and UA, while correlation with albumin and eGFR is negative. It seems that LPA concentrations have no specific circadian rhythm, therefore the time of blood donation and fasting state have marginal effect on LPA concentrations. We believe that these findings will be helpful in future application of LPA as a diagnostic marker.

## Abbreviations

ATX: Autotoxin
BMI: Body mass index
eGFR: estimated glomerular filtration rate
GLM: General linear model
HDL-C: Cholesterol of high density lipoproteins
LDL-C: Cholesterol of low density lipoproteins
LPA: Lysophosphatidic acid
LPA1–6: Lysophosphatidic acid receptors 1–6
LPC: Lysophosphatidylcholine
TAG: Triacylglycerols
TC: Total cholesterol
TP: Total protein
